# A Manual of the Principles and Practice of Ophthalmic Medicine and Surgery

**Published:** 1847-04-01

**Authors:** 


I. A Manual of the Principles and Practice of Ophthalmic Medicine and Surgery. By *T. Wharton Jones*, F.R.S. Small 8vo, pp. 570. Churchill, 1847.

II. On Cataract, Artificial Pupil, and Strabismus. By *F. A. Brett*, M.D., F.R.C.S. 8vo. pp. 89. Churchill, 1847.

III. Annales d'Oculistiqe. Tom. XVI. XVII. 1846-7.

IV. Report on the Progress of Ophthalmic Surgery for 1846, with Original Cases and Illustrations. By *W. R. Wilde*, M.R.I.A.

V. Papers on Inflammations of the Eye. By *A. Jacob*, M.D. F.R.C.S.I. (Dublin Medical Press, 1846).

We had hoped to have had M. Desmarres' work upon ophthalmic medicine in our hands before this; but the publications above cited will, in the mean time, furnish us with some interesting information to place before our readers. Mr. Jones' "Manual" is a very elaborate compilation, and will, in this age of condensing, epitomising, and manualizing, doubtless occupy the foremost place. To our own taste it is, however, needlessly extended in its earlier portions. The directions for examining the eye are frequently too anticipatory of the descriptions of its diseases, and are minuter than necessary for those, as we hope all do, who avail themselves of opportunities of practically studying this important subject, which, perhaps less than any other, is to be managed by books. Here the student has the opportunity, which so few other perverted conditions of the economy furnish him with, of *seeing* disease at its onset, during its various stages of progress, and in its different effects. This facility of inspection has, however, been disadvantageous in one respect, that of encouraging a disposition to minute and fanciful division and subdivision of the various morbid appearances, which, conjoined with the jargonic vocabulary that has been invented for the purpose of indicating these, has well nigh converted into a difficult and repulsive study, that which is really in its natural simplicity very easy, and should be attractive. Mr. Jones has farther unnecessarily enlarged his book by a long description of the various phenomena of inflammation in general. Ably as we allow this is written, it is surely time a stop were put to the practice of writers upon special subjects, taking it for granted that their readers are unacquainted with the most general principles of disease, and occupying space which should be otherwise filled, or better still spared altogether, with dissertations which find their proper place elsewhere. Among Mr. Jones' directions for examining the eye, that applicable to children seems to us a useful one.

"For the examination of the eyes in children, especially when affected with intolerance of light and blepharopasmus, considerable management is required, and even some degree of gentle force. The surgeon is to seat himself on a chair with a towel, folded long ways, laid across his knees. On another chair, on the surgeon's left hand, and a little in front of him, the nurse, with the child, sits in such a way that when she lays the child scross her lap, its head may be received on the towel, and between the knees of the surgeon, and thus held steadily. The nurse now confines the arms and hands of the child, whilst the surgeon, having dried the eyelids with a soft linen cloth, proceeds to separate them by applying the point of the forefinger of one hand to the border of the upper eyelid, and the point of the thumb of the other hand to the border of the lower, and then sliding them against the eyeball, but without pressing on it, towards their respective orbital edges. This mode of proceeding obviates the eversion of the eyelids, which is so apt to take place under these circumstances. The eyelids being thus opened, they are readily kept so during the examination, by the command, which the points of the finger and thumb, resting against the edges of the orbit, have of their borders. By this means the whole front of the eyeball is exposed, but it often happens that, to avoid the light, the eye is spasmodically turned up, so that the cornea is in a great measure concealed. By waiting a few seconds, however, enough of it will in general come into view to enable the surgeon to judge of the state in which the eye is. Having completed this portion of the exploration, there is not much difficulty in so everting the eyelids as to ascertain the state of the palpebral conjunctiva." P. 14.

We may commence with some account of

*The Ophthalmiæ.*

These Mr. Jones divides into four orders. O. Externa, Interna anterior, Interna posterior, and Panophthalmitis. The first of these is subdivided into Conjunctivitis, Sclerotitis, and Corneitis; the second, into Aquo-capsulitis, Iritis, and Crystallino-capsulitis anterior; the third, into Choroiditis, Retinitis, Vitreo-capsulitis, and Crystallino-capsulitis posterior; while the fourth (inflammation of the whole eye) is fortunate in constituting but the one order and genus. That inflammation may affect any of these textures is certain, but the practice among Ophthalmologists of describing its agency as so many isolated or separate diseases, has long seemed to us a most faulty one. This, joined to the innumerable modifications induced by diathesis detailed by other observers, renders the study of the subject unnecessarily complex, and often directs too exclusive attention to some particular structure. We are much pleased in finding so experienced an observer as Dr. Jacob, in the papers quoted at the head of this article, frequently inculcating these views. Speaking of Iritis, he objects to the term, inasmuch as other textures are as much affected, especially the retina, as is the iris, and observes :—

"The attempt to insulate or confine the inflammations of the eye to particular structures, under the names of iritis, choroiditis, retinitis, corneitis, sclerotitis, and aqua-capsulitis, hyaloiditis, &c. has not proved serviceable in practice. It looks very methodical, it appears plausible in books and lectures; but, when we come to test the matter by observation, we find many of these apparent distinctions vanish, and discover it is only a progressive inflammation of the whole organ, more conspicuous at the commencement in some particular part."

We are likewise disposed to agree with Dr. Jacob in his opinion that simple, uncomplicated, inflammation of the eye is of more frequent comparative occurrence among the lower orders than that dependent on scrofula, rheumatism, arthritis, &c., than is generally believed. Such is however not the general opinion, and certainly not that of our German brethren, who seem to revel in their multiplied subdivisions. Thus M. Cunier, in a report in the "Annales d'Oculistique," upon the diseases of the Eye observed in the province of Brabant, not content with the ordinary designations of scrofulous, catarrhal, rheumatic, syphilitic, &c., ophthalmia, combines these in every variety, so that we have the scrofuloso-catarrhal, &c. *ad infinitum;* and he is much surprised that writers, so experienced as Lawrence and Mackenzie, in noting the catarrho-rheumatismal ophthalmias, should have neglected indicating the rheumato-catarrhal. Although it is certain that, under the specific influence of these diseases, certain structures of the eye are occasionally more especially influenced than others, these are not so exclusively, nor to anything like the extent usually believed; and, moreover, the existence of any such predisposition is too hastily assumed in many cases. Still we believe that the designation of inflammations of the eye under some general appellation, derived from constitutional peculiarities, is often a very useful procedure, providing the attempt be not made to localize such classes in some one or other of the structures of the eye under the appellations corneitis, conjunctivitis, iritis, &c.

*Scrofulous Ophthalmia.*—This, Mr. Jones describes as essentially a species of corneitis—with what justice let those judge who are familiar with its various symptoms referable to the implication of other structures. It seems to be very prevalent at Brussels, for of 641 cases of eye-disease, reported by M. Cunier, 107 consisted of scrofulous ophthalmia; in 42 others the scrofulous element existed, although not predominantly, while 107 cases exhibited the consequences of prior attacks—giving a total of 256. In 103 cases, in which the ages were noted, these were less than 12 in 96, and more only in 17. Of 1782 cases of eye-disease throughout Brabant, 640 were examples of scrofulous ophthalmia or its effects. We believe the writers upon disease of the eye in all countries bear witness to a nearly similar preponderance. M. Cunier had frequent opportunities of tracing the *hereditary* influence in the production of the disease, in most cases the parents having already suffered from this or other scrofulous diseases, or other branches of the family continuing to do so. He agrees with Lugol, that the *endemic* prevalence of the disease is due rather to the life of wretched privation, mixed with intemperance, the families in which it prevails lead, than to the effect of vitiated air and crowded localities; and laborious enquiries have convinced him, that the majority of the *occasional causes* of authors have no necessary effect, scrofula only shewing itself under their influence when individual predisposition exists. Such causes, however, are very injurious to health when persistent, and no population exposed to them can rear healthy offspring.

M. Cunier, after Sichel, describes two forms of the disease; the one irritable, erethitic; the other indolent, phlegmatic, or torpid: both manifesting that predominance of the lymphatic system and irregularity of the digestive functions, so characteristic of scrofula. There were about 54 per cent. irritable, and 46 torpid, in the towns; and 33 irritable, and 67 torpid, in the rural districts of Brabant.

Mr. Jones' directions for the *treatment* of this disease are judicious. He commences with an emetic and purge, keeping up some action of the skin, if feverishness is present, by means of antimonial wine, and afterwards regulating the condition of the digestive organs by hydr. c̄ creta and hyoscyamus, with occasional doses of calomel and rhubarb or scammony. This accomplished and fever subdued, a grain or two of quinine ter. will, in most cases, seem to exert almost a specific effect, while in others iron, sulphuric acid, or rhubarb and soda, will be found useful. He recommends the occasional application of a few leeches around the eye, a measure from which, in these cases, we have seldom derived benefit, while from the practice of counter-irritation behind the ears, highly approved by him, we have seen the best results follow. As local applications he employs a belladonna lotion, or steaming the eyes with vapour impregnated with belladonna, as effectual means of relieving the intolerance of light. No mention is made of the painting the eyelid and eyebrow with iodine for this purpose—a practice frequently very successful. Due attention to the purity of the air, condition of the clothing, diet, and other hygienic circumstances, is all-important. Where the disease proves very obstinate, evacuation of the aqueous humour, repeated leechings and blisterings, a mercurial course, in conjunction with the quinine, and the continuous dilatation of the pupil by belladonna, are indicated.

*Rheumatic Ophthalmia.*—Dr. Cunier represents this as of extremely frequent occurrence among the working-classes of Brussels. Scrofula is the most ordinary combination, and catarrhal ophthalmia a very frequent one. Sudden atmospheric changes, ill-ventilated, heated workshops, and the sudden change of temperature in passing from these to the air, and travelling in exposed carriages on railways, are among the causes assigned for the production of the disease.

Dr. Jacob well observes, that the information, respecting rheumatic inflammation of the eye, to be found in ophthalmic writers is anything but precise.

" It seems to be assumed that rheumatic inflammation of the eye has its seat more especially in the sclerotic coat, apparently from a belief that rheumatism generally affects the fibrous membranes of which the sclerotic is one. It remains, however, to be proved that, in this disease, the ligaments and tendons about joints, or the fibrous apparatus of other organs, are the parts first attacked, or indeed those attacked at all; except in consequence of their connexion with the serous, synovial, or muscular structures engaged in the disease. That the sclerotic is not more the seat of this disease than the other textures of the eye, I am convinced, and the sooner the notion of its being so is abandoned the better; because it has the effect of directing the practitioner's attention from the more important consideration, that the whole of the organ is engaged, and the parts essential to vision are in great danger."—*Press.* xvi., p. 193.

This writer also comments upon the insufficiency of the diagnostic signs first attempted to be laid down with precision by Wardrop. He does not deny that such are present in many or most cases of rheumatic ophthalmia, but they are absent in others, and may exist quite independently of the rheumatic diathesis. The formation of the zone round the cornea by the minute subdivision of distinct ramifications, is found in simple as well as rheumatic ophthalmia, although often rendered indistinct by co-existent conjunctival vascularity. This zone, in acute inflammation occurring in young persons, is of a bright pink, but in older persons, or where the disease has long existed, so that the veins have become enlarged as well as the arteries, this may be changed into the brick-red, or yellowish-red, without necessarily indicating rheumatic disposition. The aspect of an eye long inflamed and in old age, is very different from that of one acutely inflamed in early life. Although the vascularity is not alone characteristic, it is generally more considerable, both conjunctival and sclerotic, than in idiopathic or simple inflammation of the iris. The cloudiness of the cornea also, described by Mr. Wardrop, is sometimes seen in simple, and is characteristic of gonorrhœal iritis; nor are the changes which occur in the iris peculiar. The agonizing, dull, remittent, orbital pain, so well described by Wardrop, is not always present, nor exclusively indicative of this form when it is.

"Although, as has been already stated, the specific nature of the inflammation cannot be recognized with certainty from the changes of structure or the symptoms, it may be apprehended from the severity of the attack, the greater degree of pain, and its extension to surrounding parts, as well as from intolerance of light and general constitutional disturbance. Neither these peculiarities, nor any other modification of symptoms, will, however, in my opinion, justify the practitioner in pronouncing positively that the disease is true rheumatic inflammation of the eye, unless there be unequivocal proof of the previous or present existence of rheumatic constitution or diathesis, as indicated by inflammation of the joints, with the peculiar accompanying fever, or that disturbance of the system called acute rheumatism; or unless there be at least transient shifting pains of joints or muscles, with brief febrile paroxysms, perspirations, lithic deposits in the urine, and general ill health. It should not be assumed that inflammation of the eye is of true rheumatic nature, because the patient may have occasionally experienced temporary pains of some of the joints or muscles. * * * * To call an inflammation of the eye rheumatic, because it appears to be situated in the fibrous membrane or sclerotic, is nothing more than an intimation that the disease is to be considered of this nature merely because it attacks that structure, and is obviously made from an assumption, unsupported by fact, that these textures are peculiarly subject to such disease. Rheumatic ophthalmia, it is said, 'frequently occurs in individuals who have never suffered from rheumatism in other parts of the body.' If so, the disease so called is not rheumatic. Rheumatic inflammation of the eye may perhaps sometimes, but not frequently, occur without inflammation of joints or other organs, but not without constitutional rheumatic disease. If that be not present, the local inflammation is destitute of the specific character." P. 196.

*Treatment.*—Mr. Jones describes rheumatic inflammation of the eye under two separate heads, those of sclerotitis and rheumatic iritis. The former, he states, may, in its incipient stages, be sometimes checked by a dose of calomel and Dover's powder, with a pediluvium at night and an aperient in the morning, following these up with small doses of nitre. If this is not successful, bleeding as also in rheumatic iritis, must be resorted to. In ordinary inflammation of the iris and other structures of the eye, Dr. Jacob is an advocate for more cautious depletion than that usually employed, believing that the most destructive effects often occur after comparatively slight inflammation, and in the less robust subjects. "After many years' experience, I find myself treating inflammation of the eye without much bleeding, and I do not think my success is less than that of practitioners who resort principally to the lancet and leeches." Bleeding is useful only at the very commencement, and should not be resorted to for the removal of the mere redness of a later period. If in ordinary inflammation this practitioner considers a modification of the usual practice desirable, he does so still more in reference to those cases connected with the rheumatic diathesis, in which depletion should, save in the robust and plethoric, be only local. Both Mr. Jones and Mr. Jacob recommend repeated doses of Dover's powder, and diaphoretic doses of tartar-emetic, occasional purgatives, as sulphate of magnesia with the carbonate, calomel and colocynth, &c. In severe cases *mercury* is required and should be combined with antimony and opium; but the same reliance is not to be placed upon this mineral as in the simple or syphilitic form of the disease. Upon the utility of *colchicum* much discrepancy of opinion prevails, and Dr. Jacob speaks somewhat doubtingly upon the subject. From *cinchona*, in debility and decayed health caused by active treatment, and in examples of frequent relapse, like most practitioners, he has derived great advantage. In those chronic and refractory cases, too, the *iodide of potassium* is given with bark, mercury, &c. as the case may be, with benefit. The ammoniated tincture of *guaiacum*, given in milk, is another useful remedy. Belladonna should be used both as a fomentation (ʒj. Ext. ad ℔j. Dec. Papav.) for the relief of pain, and as a means of maintaining the pupil dilated. Blisters are often beneficial; and, in the latter stages, the *vinum opii* dropped in the eye, affords much relief where there is a scalding pain, intolerance of light, and lachrymation.

*Gonorrhœal Ophthalmia.*—Mr. Jones enumerates only one cause as capable of producing this destructive disease, viz. inoculation with gonorrhœal matter: but we think M. Cunier's views are more correct. He classes the patients who offered themselves to his notice into several groups. 1. Instances of inoculation of the gonorrhœal matter, either from the urethra of the individual, or that of another person. 2. Those in whom it arose from the sympathy prevailing between the urethral or vaginal mucous membrane and the conjunctiva—structures very analogous in structure.

"It is a well known fact," says M. Cunier, "that persons who habitually give themselves up to venereal excesses have the edges of their eyelids red and tumid, the conjunctivæ injected, the eyes themselves sensitive and weeping upon the least excitement. This is especially the case in scrofulous persons and in leucorrhœal women. An increase of leucorrhœa, or an acute gonorrhœa is always accompanied in such with a degree of lippitudo. If it so happen that they are subjected to any cause which under other circumstances would have induced a simple ophthalmia, they will have a true ocular blenorrhœa, though not of a venereal nature."

3. In these, a sudden and almost total suppression of the urethral discharge took place—a metastasis. M. Cunier believes examples of metastasis to be extremely rare, and has indeed never met with an absolute suppression. "What has been usually termed gonorrhœal ophthalmia by metastasis, appears to be but a sudden exaggeration of the sympathetic affection of the conjunctiva, and this to a degree sufficient for inducing a revulsion, capable of producing a quasi-cessation of the original flux, which may become as intense as ever when the ocular inflammation has diminished." In the same way, a violent purging may temporarily diminish the ocular or genital flux. In support of this opinion may be adduced the cases of Fischer and Ribes, in which the ocular and genital fluxes alternated in severity. So too, in a case of ophthalmia induced by inoculation for pannus, which suddenly acquired intensity, the urethral discharge became notably diminished.

4. It is familiarly known that the disease may be communicated by bringing the matter from a diseased eye in contact with a sound one. 5. And among the cases noted were some in which the disease had been conveyed to certain individuals shut up in a confined space with one suffering under it, and that by *miasmatic* agency only.

Professor Hairion of Louvain, in an elaborate essay on this disease, recently published in the "Annales," announced as an invariable pathognomic sign of its existence, the presence of a small, rounded or oval, subcutaneous, painful tumour, seated just anterior to the ear of the affected side, and produced by an enlargement of a lymphatic gland. M. Sichel having, therefore, turned his attention to the subject, likewise observed the "*pre-auricular bubo*" in many cases; but, alas! for the hasty generalization of his *confrere*, he likewise discovered it in other cases of disease of the eye, partaking in no-wise of a venereal character, it being in fact but one of the manifestations of a scrofulous disposition.

We are glad to find that Mr. Jones countenances the early employment of strong local irritants to the eye in this destructive class of diseases. The profuse venæsections, once so exclusively depended upon and still resorted to by some to the neglect of the nitrate of silver in substance or in ointment, have lost many an eye which might have been saved.

We had intended considering the various ophthalmiæ *seriatim*, but want of space compels us to limit our further notice to *Infantile Purulent Ophthalmia.* This disease is of very frequent occurrence at Brussels, a fact which Dr. Cunier endeavours to account for by the great prevalence of leucorrhœa amongst the lower ranks of females of that city. He states the results of all the researches he has made upon the subject, confirm the conclusion of Professor Cederschjoeld, of Stockholm, that when the head is long passing through the passages of a leucorrhœal woman, the escape of the child from this ophthalmia is the exception. This opinion of the frequent agency of leucorrhœa is held, we believe, by most observers; but we altogether doubt its correctness, not only on the ground stated by MM. Vidal and Velpeau, that the child enters the world with its eyes closed and with the eyelids folded over each other; but because the affection would be immeasurably more common than it is, were a cause of such frequent and, in large towns, almost constant existence competent to its production. M. Cunier, indeed, recognizes other causes, and thus distributes them in 218 cases; 41 were attributed to the ordinary causes of catarrh; 15 to exposure to light, accidents, or want of cleanliness; 18 to the contact of the eyes with gonorrhœal matter; and 144 to their contact with leucorrhœal discharge. Of 113 cases, the disease manifested itself in 19 on the third day; in 35 on the fourth; in 29 on the fifth, and in 20 on the sixth. Founded upon his views of the leucorrhœal origin of the ophthalmia, M. Cunier recommends that the eyes of all new-born infants be washed directly after birth with water. To this we see no objection, providing this contain no soap, although we feel convinced it is a nugatory practice, as it is inconceivable that removable matter, capable of producing so destructive a disease, should continue in contact with the delicate conjunctiva and produce no effect, in most cases, for several days; but when, as a prophylactic, M. Cunier recommends the use of corrosive sublimate, or chloride of lime lotions directly after birth, we think he risks inducing irritation, which else might never have occurred. He states, however, that in the case of the infants of women suffering from leucorrhœa or gonorrhœa, almost all the Belgian practitioners now follow this practice.

Many have doubted the contagious property of the matter secreted from the infant's eyes in this disease, and with some show of reason, seeing the rarity with which the mother becomes affected, even in those classes in which cleanliness and precaution cannot be adduced to explain her immunity. M. Cunier, however, among the cases we have adverted to, met with eight instances of this. In one of these, the towel which had been employed for the infant communicated the disease to the mother and two of her children. In three other cases, the disease was also propagated to the mothers, and in one to the father. It may be propagated also miasmatically in crowded wards of foundling hospitals.

Few diseases are more satisfactory in respect to the results of treatment than this, the affection seldom proving unmanageable, save in the case of the very poor attended by ignorant midwives. The practitioner being in attendance upon the mother has the opportunity of witnessing the disease at its very onset. Indeed, he may mistake a sticking together of the eyelids, a slight vascularity of their conjunctival lining, and a watery discharge from the eye, for the disease in question, and unnecessarily resort to active remedies. It is better, therefore, to wait until the discharge puts on its purulent character, or until the upper eyelid becomes somewhat congested or tumefied, before we resort to these, pretty certain as we are of overtaking the malady shortly. When assured of its nature, we always ensure the mother's diligent attention, by informing her that the loss or preservation of the eye entirely depends upon the rigid observance of the simple directions furnished her, which consist in ordering the injection into the eye (after previously throwing water into it), by means of a glass syringe, of a portion of a collyrium, formed of gr. v. of alum or gr. ij. of arg. nit. *every hour,* day and night, until the discharge much abates, and then proportionally seldomer. The woman is usually frightened at introducing the substance *into* the eye, and, unless shewn how to proceed, will apply it only to the exterior; but, soon perceiving the benefit which accrues, she is encouraged to proceed. Rarely do we find it necessary to resort to leeching, blistering, or any other procedure, save the administration of a mild alterative purge. So uniform and speedy is the success which attends this practice, that we had been long accustomed to give an unhesitating promise of recovery upon its fair adoption; but, from a case or two of late, of no remarkable severity in appearance, indeed quite the contrary, occurring in the enfeebled children of sickly parents, we have learned more caution in stating the force of the conviction we feel.

Mr. Wilde, speaking of this disease, says—

"Having constantly remarked an extensive state of ulceration in the conjunctiva of the upper lid, in the severe forms of this disease, I now generally evert the lid to examine its inner surface as soon as a case presents itself; and I have several times succeeded in cutting short the disease by at once applying a strong solution of nitrate of silver to this part alone. We beg to call the attention of ophthalmic surgeons to this subject."

### *Congenital Tumours of the Conjunctiva.*

Mr. Wilde has commenced publishing, in the pages of our able contemporary, the "Dublin Quarterly Journal," a series of Reports on the Progress of Ophthalmic Surgery. Although of opinion that the class of literary production in the shape of "Reports," "Abstracts," and the like, is generally a very wearisome species of reading, rising little above the dignity of an index *raisonné* of the contents of the various periodicals either in interest or utility, we can speak of the present one as a favorable exception, in not being framed in so condensed a manner as to render perusal painful and retention impossible, and in being interspersed with sound criticism and observations, indicative not of the mere compiler, but of the original enquirer. It contains an interesting case of Congenital Tumour of the Conjunctiva, which we proceed to transcribe.

"We were lately sent for by Capt. B., of whom we had no previous knowledge, and who was then confined to his room from what we were informed was 'a severe cold and inflammation of the eye.' On arriving at his hotel, we fouud him labouring under great intolerance of light, lachrymation, and some œdema and redness of the lids of the right eye. Being a person of rather eccentric manners, he refused to give any history of his disease, or describe his own feelings and symptoms, until we had pronounced upon his case. On examination we found the entire conjunctiva highly injected, and two large vascular masses projecting from the surface of the globe; one, the lesser in size, and least apparent, protruded from under the upper lid, just beneath the situation of the lachrymal gland; it was of a deeper pink than the rest of the conjunctiva, and appeared firm and unyielding. The second and most remarkable tumour was, in its then condition, about the size of a horse-bean, placed transversely on the globe, one-third of it lying in the cornea, the other two-thirds occupying the outer side of the sclerotic. Like that which protruded from beneath the lid, this was of a deep, pink hue, and slightly lobulated on the surface, not unlike a half-ripe raspberry. A gush of scalding tears, attended with increased pain and photophobia, followed immediately this examination. We at once pronounced them to be *congenital tumours* in a state of inflammation, and such they were; that which encroached on the cornea had several light-coloured hairs growing from its surface. These generally lay quiescent between the palpebral aperture, or projecting slightly over the edge of the lower lid, seldom caused any inconvenience. The largest had, however, two days before, turned up under the superior lid, and gave rise to all the symptoms we have described. Its removal caused them to subside almost immediately. The case is interesting and instructive on account of its having been first seen during an attack of inflammation, or, more properly speaking, inflammatory irritation, and from the possibility of its being thus mistaken for a sudden morbid growth. What first awakened suspicion, the moment the lids were separated, was the fact of the tumour being covered with cutaneous epithelium, which, as in cases of xeroma, gave it the appearance of being oiled or varnished, so that the tears did not flow over it, and moisten all its surface, but lay upon it in detached globules. This cuticular character is peculiar to all those growths from which hair grew which we have examined. We have since seen the eye in a quiescent state, and find our conjectures were correct."

### *Cataract.*

Although several of Mr. Brett's observations will be found useful to the young operating surgeon, we are at a loss to discover sufficient novelty in these to call for the publication of a new work upon "Cataract, Artificial Pupil, and Strabismus." Indeed, little is to found in it that is not far more amply detailed in most other works—Mr, Jones' Manual among the rest. The *statistics* of various operations for cataract have been so variously stated by different observers, as to have little claim to our notice. Mr. Brett refers to some of these discrepancies; but we should have better like to have seen a detailed account of the results of his own practice in India, which we believe some recent advertisements stated to have been both extensive and remarkably successful. Mr. Wilde furnishes us with an abstract of Dr. E. Jäger's account of his father's practice at Vienna. It seems, between 1827 and 1844, he operated upon 1011 cataracts: 728 times by superior extraction, 9 by inferior, 50 by partial extraction; 129 by depression, and 87 by absorption. Of this number, 63 were unsuccessful; the proportion of those who have irrecoverably lost their vision to those who have been successfully operated upon are, in extraction $4\frac{3}{4}$ per cent.; in depression 16; and in breaking up 8 per cent. This is indeed high testimony in favour of extraction; but Mr. Wilde considers the tables as very defective in some necessary details. Mr. Brett remarks, that extraction is much oftener preferred in Germany and England than in France, which he attributes to the absence of special eye infirmaries in the latter country, and a consequent want of opportunity of practically examining into the merits of this operation. In his own practice, Mr. Brett observes, "In India, in the healthy and robust natives of the Western provinces, I have found extraction the most successful; whilst, in the lower provinces of Bengal, where the inhabitants are more feeble, I have not found extraction so successful; but, at the same time, it must be admitted, that we have not many opportunities of watching the result for a long period after the operation." The following is his general summary of the advantages and disadvantages of the respective methods.

"By extraction, the obstruction to vision is entirely removed. It is not usually painful, is seldom followed by internal inflammation, neither the ciliary nerves nor the ciliary vessels are wounded, the whole of the interior of the eye is untouched, the retina, the choroid, ciliary circle, &c.; and, lastly, secondary capsular cataract is less liable to follow this than any other operation. On the other hand, the iris may be cut, and the vitreous humour may escape; if the wound of the cornea does not unite by the first intention it may ulcerate, and the iris will prolapse; inflammation and suppuration of the globe may be the result—at all events, the pupil will become closed.

"In depression, purely so called, the vitreous humour does not escape, the cornea preserves its transparency, there is no chance of prolapsus iridis, or of its excision, and the operation may be repeated if requisite; but, on the other hand, the evils of the operation are acknowledged by all, and are numerous. The lens acts as a foreign body, and often causes much irritation at the bottom of the eye—it is liable to re-ascend: the operation is frequently followed by membranous cataracts, by iritis, deep-seated pain, and general nervous irritation. The needle necessarily penetrates the choroid, the retina, the vitreous humour; and the ciliary processes are at least somewhat disturbed: inflammation is as frequent as after extraction. The wound of the sclerotic, the choroid, the retina, and the vitreous body, does not necessarily produce more pain or injury than that of the cornea, when carefully done. With due precautions it is easy to avoid wounding the nerves or vessels, or the ciliary body. When the capsule of the crystalline is well cut up, there will be less chance of secondary membranous cataract: in short, much depends upon the skill and address of the operator to avoid most of the evils of depression, to prevent the lens rolling round the needle, or falling into the bottom of the anterior chamber, and to place it flat-wise at the bottom of the vitreous humour." P. 51.

From this extract it is not very easy to decide which operation the author prefers. Mr. Jones speaks far more precisely in the following passages.

"By the operation of extraction, the cataract is removed wholly and at once from the eye, and very good vision restored: but the operation is a nice, if not a very difficult one, and liable to the occurrence of the various untoward circumstances above mentioned, by which its success may be marred.

"The operation of displacement, which may be performed in the same cases as extraction, is neither so nice or so difficult, does not expose the eye to the same risk of immediate destruction, and though the cataract is apt to return to its former place, the operation may be repeated; but though displacement may have succeeded as an operation, and vision be restored, the eye is not so safe as after successful extraction, but is liable to become affected with internal inflammation, which ends in amaurosis.

"Extraction thus possesses a decided advantage over displacement, and is therefore generally preferred, except when the unfavourable complications above-mentioned (viz. unsteadiness on the part of the patient, dyspnœa, overhanging orbit and eye-brow, narrow palpebral fissure, very sunk or prominent eye-ball, unhealthy, small, or flat cornea, and consequently small anterior chamber, synechia, small and undilatable pupil, and especially a dissolved state of the vitreous body and its connexions) exist. The degree of softening of the vitreous body requisite to admit of safe displacement of the lens is not so great as to forbid extraction, but of course, if, in the cases in which the vitreous body is so much dissolved that the displaced lens is apt to float up again, displacement be contra-indicated, extraction is much more so. All other things being equal, it might perhaps be laid down as a general proposition, that, in the very cases in which displacement admits of being most readily and safely performed, extraction is less safe; whilst, on the other hand, in the cases in which, in consequence of the soundness of the vitreous body, extraction is most safely and easily performed, displacement is least so.

"As the cases for which division is best fitted are different from those in which extraction or displacement is indicated, there is no comparison to be made between them. It is, however, to be observed, that a combination of division and extraction is sometimes had recourse to in cases of common lenticular cataract of old people. The object of having recourse to this compound operation is that the lens may, by solution and absorption of its soft exterior parts, be reduced to its hard nucleus, which, in consequence of its small size, will admit of being extracted through a small section of the cornea."—*Manual*, p. 292.

M. Laugier described, in a recent number of the "Annales d'Oculistique," an operation for cataract, which he terms by "*Aspiration*" performed by means of a Scarpa needle rendered tubular and adapted to Anel's syringe. This is carried completely into the crystalline, care being taken not to injure either capsule, and, in the case of soft or partially soft cataract, the opaque body is removed by exhausting the tube of the knife of the air it contains by means of the syringe attached to its handle. The priority of the contrivance has been contended for by subsequent correspondents of the Journal; and it is certain that suction has, in different manners at different periods, been applied to this purpose. Ingenious as M. Laugier's modification is, we fear it is destined to be of little practical utility.

### *The Operation for Strabismus.*

Mr. Brett, as the result of his own practice in India, and his observation of that followed upon the Continent, offers the following suggestions.

"1. To use the scissors always in preference to the knife and director, because by the former you can more readily divide every contiguous fibre both above and below. To the practised hand the point of the scissors grates along the hard fibrous sclerotic coat, and the operator is quite certain that he cuts every muscular fibre. 2. Never to use the hook to claw out the eye, which has always appeared to me at least an unsightly instrument, and somewhat painful, but chiefly because it must be entrusted to the hands of an assistant, who, unless he possess the delicate touch of an artist, and the steadiest hand, may rotate the eye not precisely in the horizontal line. If he give the instrument the least obliquity, he disconcerts the operator in his search after the muscle he has to divide, and he finds it either above or below where he expects it. 3. The operator should have the lids and eye-ball completely under his command; and this is effectually attained by the speculum of Velpeau. The ordinary speculum often slips, and confuses or obscures the operation; so do the fingers. This cannot be the case with the speculum of the Professor of La Charité. I have always operated with the scissors, and I have never met with an instance of the return of the squint; but I have never seen the inconveniences of that part of the duty which devolves on the assistants so happily obviated as by the mode I witnessed in Paris, adopted by Professor Velpeau. I now proceed to describe the operation upon the principles by which we should be guided in all surgical operations, viz. with the least possible pain, the greatest facility and rapidity, in the safest and most effectual manner.

"The surgeon must bear in mind the insertion of the muscles into the sclerotic three lines and a half from the circumference of the cornea. The patient is placed before a clear light; the opposite eye is obscured; the head supported by an assistant; the lids separated by the elastic wire speculum (the blephareirgon). The surgeon seated in front seizes the conjunctiva, together with the attachment of the muscle itself by means of the forceps, near its insertion into the sclerotic, about four lines from the margin of the cornea. This gives the operator complete controul over the eye, and by it he is enabled to draw the eye outwards. Immediately after this, he grasps the belly of the muscle with a second pair of forceps. This latter is entrusted to an assistant. The muscle is thus raised and stretched between two pairs of forceps. Its section between the two points of transfixion is now an instantaneous affair. The smooth and bluish-white sclerotica shines beneath the incision, and the operator satisfies himself that every fibre is divided, carrying the scissors above and below until the cornea assumes its central position, and the patient can turn the eye to the opposite side, yet is unable, by any effort, to squint. Finally, the tendinous edge of the muscle grasped by the first pair of forceps, together with some loose portions of conjunctiva, is excised by a stroke of the scissors. This last procedure prevents a very common occurrence, viz. a fungoid granulation, formed from the ragged edge of the tendinous extremity of the muscle, and some cellular tissue beneath the conjunctiva. The eye is cleansed from blood by sponging with cold water. Obscure the opposite eye for some days. Let the patient use the eye which has been operated on in a moderate light. This prevents adhesion and contraction, which might cause a return of the squint." P. 80.

We cannot quote any observations calculated to facilitate the performance of this operation without declaring that it has been undertaken, during the last few years, with a recklessness and charlatanerie truly disgraceful to our profession, whether we consider the great fundamental principle which should always guide the surgeon, of never performing an unnecessary operation, or the disastrous consequences which have sometimes followed its apparently trifling infringement in so simple a case as this.

In conclusion, we may observe that the works of both Mr. Jones and Mr. Brett are freely illustrated with wood-cuts, and that of the former gentleman contains a good etymological glossary, as indispensable an accompaniment in the study of the diseases of the eye as is a dictionary for the mastery of a foreign language. Certainly single terms, however barbarous, are better than circumlocutions, but surely they are unnecessarily multiplied in ophthalmology, *e. g. Amphiblestroiditis* for Retinitis; *Blepharoblenorrhœa,* the first stage of puro-mucous inflammation of the conjunctiva; *Blepharophthalmia,* the same fully formed; *Dacryo-cysto-blenorrhœa,* discharge from the lachrymal sac, &c. &c.

